# Regulating the Market in Human Research Participants

**DOI:** 10.1371/journal.pmed.0030330

**Published:** 2006-08-01

**Authors:** Trudo Lemmens, Paul B Miller

## Abstract

Lemmens and Miller critically examine "finder's fees" and other recruitment incentives issued to physicians for successfully referring patients to clinical trial investigators.

In the past couple of years, several investigations by the Office of the Inspector General (OIG) of the US Department of Health and Human Services (DHHS) have drawn attention to the use of recruitment incentives in research. In 2003, Toronto's *Globe and Mail* reported that the pharmaceutical company Biovail became the subject of an investigation because it paid $1,000 per patient to American physicians who managed to renew prescriptions of its new drug Cardizem LA for at least 11 patients. The company argued that the payments were a reward for data gathering for post-marketing research [1]. Less successful physicians received only $250 per patient. In 2005, the OIG started an inquiry into payments made by Advanced Neuromodulation Systems to physicians who implanted a pain-management device in their patients for a five-day trial [2]. According to the *Wall Street Journal*, those who managed to implant the device in at least five of their patients received $1,000 for “data collection and management of the trial process.”

The practice of paying research participants is inadequately regulated.

Although the OIG has not yet released results of either investigation, it previously documented in 2000 other examples of troubling practices in a special report on the use of recruitment incentives [3]. Among them were an Internet advertisement by a family medical practice, highlighting their ability to quickly recruit patients for drug trials and post-marketing studies using two full-time research coordinators and their computerized patient database; and an industry article on recruiting patients into studies, recommending that researchers secure “an endorsement by your well-respected newspaper reporter or TV news anchor” to generate “more phone calls needed to fill studies.”

These examples have to be understood in the context of the pervasive commercialization of medical research. General concerns relating to commercialization have received considerable attention. The practice of paying research participants has also been extensively debated [4–8] but remains inadequately regulated [9]. Much less attention has been paid, however, to issues surrounding “finder's fees” and other recruitment incentives issued to physicians for successfully referring patients to clinical trials investigators. This article aims to partly fill this void.

## Recruitment Incentives in Context

The increasing prevalence of recruitment incentives is directly related to growing competition between research sponsors. More pharmaceutical clinical trials are being undertaken than ever before. US statistics indicate industry investment of increasing magnitude in clinical trials. As of 1999, more than 450 heart, cancer, and stroke drugs were under development in the US, and a further 191 were under development for Alzheimer disease, arthritis, and depression [10]. Pre-clinical trials are also on the rise. In 1998, there were 3,278 drugs in pre-clinical testing in the US, an increase of 26.8% from 1995 [3].

Similar trends are evident elsewhere. Health Canada has indicated that it reviewed “over 800” applications for approval to proceed with clinical trials in 1998, and that it has witnessed an average 20% annual increase in clinical trials conducted in Canada [11]. These trends partly explain heightened demand for research participants. Another contributing factor is the trend toward larger trials. According to the OIG, clinical trials supporting new drug approval applications averaged 4,237 participants in 1995, an increase of 2,916 in one decade [3].

Speed of testing is as crucial as recruiting sufficient numbers of patients. Thomas Bodenheimer mentions, without providing a source, that a single day's delay in getting a drug to market costs $1.3 million [12]. Claims associated with costs of drug development merit careful scrutiny since they are often used as a rhetorical tool to argue for faster approval times or to justify the high price of pharmaceuticals. However, it is fair to presume that delays have financial repercussions. At a 2003 conference, Neil Maresky, vice president of scientific affairs at Wyeth, stated that problems in patient recruitment are “the biggest delaying factor in clinical trials” [14]. Patient recruitment is thus a crucial challenge, one that industry is attempting to address through use of financial incentives.

## Payments to Health-Care Professionals

Industry research sponsors are increasingly paying finder's fees to health-care professionals to encourage them to recruit patients. Finder's fees can be defined as payments to physicians, nurses, or other health-care professionals for the mere recruitment of research participants. Reports suggest finder's fees ranging between $2,000 and $5,000 per patient are common [15], although it is not always easy to distinguish the reward for the recruitment of patients from remuneration for clinical activities that are part of the research.

These incentives may partly explain the increasing involvement of community-based physicians in clinical research. Between 1988 and 1998, the number of community-based physicians participating in research in the US increased by 60% [16]. Sponsors target community-based physicians because their patient bases are seen as an untapped reserve of potential research participants. Academic researchers, in turn, now feel they must compete with community-based physicians for recruitment incentives, either for personal gain or to pay researchers' salaries.

Direct payments to researchers, often with bonuses for fast recruitment, are not the only tools of the trade. Sponsors may offer other incentives, including authorship priority, paid consulting work, and further research sponsorship [12,17]. For this reason, we use the wider term “recruitment incentive” rather than “finder's fee” to discuss the general ethical and legal issues associated with various practices used to entice physicians into recruiting patients.

## Concerns Raised by Recruitment Incentives

### Interference with physicians' judgment

A general concern is that recruitment incentives will encourage physicians to act contrary to their fiduciary obligations to their patients. The prospect of considerable fees for referrals may interfere with the judgment of physicians trusted by patients to act in their best interests. A related concern is potential interference with consent processes. Fraudulent behavior can be dealt with under criminal law [18]. But other forms of influence are likely to be more subtle and therefore harder to control. Researchers who know that enrollment of an additional patient will bring a $20,000 bonus may be tempted to find a way to convince that patient of the “advantages” of participation.

### Patient safety

Safety is another important concern: financial interests associated with the recruitment of patients may encourage researchers to disrespect inclusion and exclusion criteria, putting patients at risk. Misconduct at a VA (Veterans Affairs) hospital in Albany, New York, highlights this concern [19,20]. Federal officials launched a criminal investigation against two researchers involved in a cancer study at the hospital in which at least five patients died [21,22]. An inspection report by the US Food and Drug Administration (FDA) concluded that patients' medical records were altered in at least five experimental drug studies, enabling veterans to be enrolled in studies for which they were either too sick or too healthy to qualify [23]. The hospital reportedly received a fee of $5,000 for each patient enrolled [23], and some individual investigators are reported to have received undisclosed recruitment incentives [19]. The junior researcher pled guilty to criminally negligent homicide as well as fraud and was sentenced to six years in prison [24]. The senior researcher was not charged, but the FDA initiated disqualification proceedings against him on September 22, 2004 [25]. As of June 5, 2006, the case was still open (confirmed in a phone call to the FDA's Division of Scientific Investigations on June 5, 2006). Safety concerns may be amplified with the involvement of community-based physicians, who may lack experience in research, who may be overburdened, and who work more in isolation, perhaps making it harder to critically evaluate and discuss research benefits and risks with other professionals.

Patients have become de facto market products.

### Erosion of public trust in clinical research

More general concerns relate to the public interest in clinical science. In a competitive environment, commercial sponsors enjoy significant control over research. Careful selection of patients and development of research methodology, combined with selective publication, may lead to the approval of minimally effective and potentially harmful drugs. A host of recent controversies indicate how pharmaceutical sponsors have engaged in the selective publication of results, the manipulation of data, the use of ghost authors, and, allegedly, the fraudulent promotion of off-label prescription, on the basis of questionable research [26]. Erosion of scientific integrity risks the health of future patients and places undue burden on publicly funded health care. Further, recruitment incentives are implicated in the distortion of research agendas and priorities [27]. Researchers who conduct publicly funded research may encounter difficulties recruiting patients because they cannot offer significant recruitment incentives. As a result, valuable research may be neglected for research of sometimes questionable scientific importance and clinical value.

## Existing Controls on Recruitment Incentives

Few would argue that patients in trials should be treated as commodities, but patients have become de facto market products, while “market controls” are neither clear nor sufficiently stringent.

Various organizations have taken a stand against certain recruitment practices. The American Medical Association's Council on Ethical and Judicial Affairs stated unequivocally in one opinion that “offering or accepting payment for referring patients to research studies (finder's fees) is unethical” [28]. In a report on finder's fees, the council clarifies the basis for its recommendation, and seems to widen its ambit. It states that “any kind of compensation in return for the referral of patients” is unethical [29]. An earlier general opinion on conflicts of interest indicates, however, that remuneration that is “commensurate with the efforts of the researcher on behalf of the company” is acceptable [30].

The Canadian Medical Association has issued a statement on relations with the pharmaceutical industry that refers to finder's fees [31]. But it fails to clearly prohibit them or to mandate their disclosure, and essentially diverts the issue to institutional review boards (IRBs). Several academic institutions offer more direct guidance, expressly prohibiting finder's fees [32–37]. But since most clinical drug trials now take place outside of academic institutions, such guidance has little impact on the practice. It is also not clear whether academic institutions have thorough control over the research practices in their institutions, even when they have such policies in place.

IRBs are currently neither sufficiently regulated nor independent.

Physicians should, however, be aware that legal and regulatory sanctions may be imposed on them for accepting recruitment incentives. Significant private liability may arise. If physicians purposefully misinform or fail to adequately inform patients of their financial interests, they expose themselves to tort liability for battery or negligence. *Moore v. Regents of the University of California* [38] suggests that non-disclosure of financial interests by a physician can give rise to a cause of action for breach of fiduciary duty, although a more recent Florida District Court rejected the argument that physicians have to disclose financial interests [39].

Other serious legal consequences, including criminal charges, are also possible [40]. Most US states [41] and some Canadian provinces [42] have statutory frameworks on professional misconduct, with provisions that could apply to research activities. The US federal “anti-kickback statute” also makes it a felony to induce referral of patients covered by federal health-care programs [43]. These statutes generally prohibit the offer or acceptance of rewards for referring patients to health-care providers or facilities. The general terms in which such statutory provisions are constructed suggests that they could readily be applied to sanction the offer or receipt of a broad range of recruitment incentives used in clinical research [40]. The *California Business and Professions Code*, for example, prohibits the “receipt, or acceptance…of any rebate, refund, commission…or other consideration… as compensation or inducement for referring patients, clients, or customers to any person” [44].

In the US, regulatory authorities have also laid charges under the federal False Claims Act in cases where financial interests were not disclosed to granting agencies, and where researchers violated regulations, compliance with which was a condition for funding [45]. In Canada, significant case law has expanded the potential scope of application of general Criminal Code fraud provisions [46]. Canadian physicians who fail to disclose financial interests in referrals or other forms of advice to patients expose themselves to possible criminal prosecution for fraud, and when the patient's treatment suffers as a result [40].

## Recruitment Incentives and Regulatory Reform

The problems raised by finder's fees cannot be resolved by focusing exclusively on sanctioning the individuals who may accept them. They ought to be addressed as part of a broader institutional and regulatory reform effort designed to address weaknesses in research governance.

Such reforms ought to include specific institutional and regulatory guidance on broad conflict-of-interest issues. Strict but narrow rules on finder's fees may fail to protect participants from the influence of conflicts arising from other arrangements that are harder to detect and control. Several organizations have come up with sensible recommendations to strengthen conflict-of-interest rules. A task force of the Association of American Medical Colleges issued two important reports on investigator [47] and institutional [48] conflicts of interest. The task force strongly recommends that institutions separate the financial management of research from their conduct and oversight, and, further, that they establish independent conflict-of-interest committees. With regard to individuals, it recommends that institutions introduce a rebuttable presumption that individuals holding a significant financial interest in a study may not participate in its conduct.

The DHHS has also recommended the establishment of specialized institutional conflict-of-interest committees in a guidance document: “Financial Relationships and Interests in Research Involving Human Subjects” [49]. If the recommendations of the Association of American Medical Colleges and the DHHS are acted upon, they may go a long way to address concerns about conflicts of interest within academic institutions. However, it is unlikely that the adoption and enforcement of more stringent institutional policies will alone satisfactorily address concerns generated by recruitment incentives.

Regulatory agencies and institutions rely too much on IRBs to evaluate and control conflicts of interest. While the development of specialized conflict-of-interest committees within academic institutions would add a layer of more focused protection, IRBs would presumably still play the central role in protecting research participants from the ill effects of conflicts of interest. Unfortunately, while IRB review can deal with small-scale, specific conflict-of-interest issues, IRBs themselves are currently neither sufficiently regulated nor independent to fulfill their important function.

Various reports clearly indicate that the IRB system is currently facing considerable challenges [50–52]. One of the core problems with regard to IRB review of conflicts of interest is the significant conflicts faced by IRBs themselves. While IRBs have an important public-policy mandate (protection of human research participants), they are deficient with respect to some basic principles of administrative law [53]. Academic IRBs often lack independence or suffer the perception of bias because of the interests of their host institutions in the approval of research. The increasing reliance of academic institutions on private sponsors augments concerns about direct or indirect institutional pressure on IRBs. Many contract research organizations have set up internal IRBs, which suffer from similar conflicts of interest. Further, for most clinical trials involving community-based physicians, commercial IRBs are employed. The legitimacy of the review provided by these IRBs is undermined by an inherent conflict of interest, given that they are paid to make a decision that has an immediate impact on the financial interests of their clients [53,54]. Improvement of the IRB system, for example through the establishment of IRBs with a strong governmentally controlled mandate and exclusive jurisdiction [53], is a logical first step in dealing with problems relating to the increased commercialization of medical research.

There are, however, other solutions that are more radical and simpler. One option is to establish an independent national institute for drug testing, to ensure the reliability of the data supporting drug approval [55,56]. Drug companies seeking to have a drug approved would submit the drug for testing to the institute, which would then negotiate a research protocol with the sponsor. The research would be contracted out to a qualified independent drug-assessment center, which would negotiate contractual terms and matters of data publication with the institute. Other proposals include the establishment of a three-pronged independent drug regulatory authority that would not only control clinical trials and post-marketing studies more directly, but also control drug promotion and publicity [57].

Proposals such as these would do much to safeguard the independence of research. They could also provide a check on competition for research participants. Strict guidelines on recruitment of, and payments to, patients could be established for these drug assessment centers, and clinical trials could be better coordinated. The number of industry-driven exploratory trials—often aimed at coming up with “good data” to support applications and marketing—would also likely decrease. More rigorous review of the merit of trials, and the resulting decrease in their number, could also help alleviate industry concerns. Centralization of patient recruitment and reduction in recruitment competition might make it easier to recruit patients for valuable research, thus also reducing pre-approval time windows. Since these proposals require a thorough overhaul of current drug-regulation structures, and since they will not occur tomorrow, it is important that regulatory and professional agencies immediately investigate other measures, including those recommended above.

## Conclusion

We note one problem raised by our recommendations. They do not address the problem of jurisdiction shopping. Strengthened regulatory structures in North America have resulted in clinical trials being moved even more often to middle- or low-income countries, where recruitment and other research-related costs are cheaper, where regulations either do not exist or may not be adequately enforced, and where research participants are even more vulnerable [58]. These developments are a real cause for concern. Our recommendations for a more stringent regulatory review in the North American context will hopefully inspire others to look at strengthening the national regulatory regimes in other countries and at developing and enforcing international standards for recruitment in clinical trials.

## Abbreviations

DHHS: US Department of Health and Human Services
FDA: US Food and Drug Administration
IRB: institutional review board
OIG: Office of the Inspector General
