# Correction: Regulating the Market in Human Research Participants

**DOI:** 10.1371/journal.pmed.0030447

**Published:** 2006-10-31

**Authors:** Trudo Lemmens, Paul B Miller

In *PLoS Medicine*, volume 3, issue 8: DOI: 10.1371/journal.pmed.0030330

Reference 13 was mistakenly not cited in the main article. It should have been added as a reference to the sentence: “Claims associated with costs of drug development merit careful scrutiny since they are often used as a rhetorical tool to argue for faster approval times or to justify the high price of pharmaceuticals [13].”

